# Ecological Location of a Water Source and Spatial Dynamics of Behavior Under Temporally Scheduled Water Deliveries in a Modified Open-Field System: An Integrative Approach

**DOI:** 10.3389/fpsyg.2020.577903

**Published:** 2020-12-18

**Authors:** Alejandro León, Varsovia Hernández, Ursula Huerta, Carlos Alberto Hernández-Linares, Porfirio Toledo, Martha Lorena Avendaño Garrido, Esteban Escamilla Navarro, Isiris Guzmán

**Affiliations:** ^1^Comparative Psychology Lab, Centro de Estudios e Investigaciones en Conocimiento y Aprendizaje Humano, Universidad Veracruzana, Xalapa, Mexico; ^2^Facultad de Matemáticas, Universidad Veracruzana, Xalapa, Mexico; ^3^Laboratorio Nacional de Informática Avanzada, Xalapa, Mexico

**Keywords:** integrative ecological-parametric approach, recurrence plots, entropy, modified open field system, spatial-dynamics of behavior, time-based schedules

## Abstract

It has been reported in non-contingent schedules that the variety of patterns of behavior is affected by the temporal variation of water deliveries. While temporal variation is accomplished by delivering water at fixed or variable times, spatial variation is usually accomplished by varying the number of dispensers and distance among them. Such criteria do not consider the possible ecological relevance of the location of water dispensers. Nevertheless, it is plausible to suppose that the intersection of the programed contingencies (e.g., time-based schedules), the ecological differentiated space (e.g., open vs. closed zones), and the relative location of relevant objects and events (e.g., location of the water source—peripherical vs. center zone) could set up an integrated system with the behavioral patterns of the organism. In the present study, we evaluated the eco-functional relevance of two locations of the dispensers upon behavioral dynamics in Wistar rats using fixed and variable time schedules in a modified open-field system. In Experiment 1, three subjects were exposed to a fixed time 30-s water delivery schedule. In the first condition, the water dispenser was located at the center of the experimental chamber. In the second condition, the water dispenser was located at the center of a wall of the experimental chamber. Each location was present for 20 sessions. In Experiment 2, conditions were the same, but a variable time schedule was used. Routes, distance to the dispenser, recurrence patterns, time spent in zones, entropy, and divergence were analyzed. Our findings suggest a robust differential relevance of the location of the dispensers that should be considered in studies evaluating behavioral dynamics. Results are discussed from an integrative, ecological-parametric framework.

## Introduction

In behavioral science, two approaches to the analysis of behavior have taken place: the arbitrary and the ecological (Timberlake, 1990). The *arbitrary approach* is characterized for its emphasis on the systematic variations of temporal parameters of stimuli and its effect on the rate of an *arbitrary response* (Skinner, 1938; Ferster and Skinner, 1957; Schoenfeld et al., 1972). On the other side, the *ecological approach* is characterized by its emphasis in the study of behavior in a context in which stimuli and responses have ecological relevance [e.g., water-seeking behavior, exploratory behavior, etc; Maier and Schneirla (1964)]. Even when the arbitrary and ecological approaches were antagonist with respect to various topics, some behavioral researchers influenced by biological sciences have been interested to find a common ground among the arbitrary and ecological perspective, taking advantage of the strengths of both approaches (Timberlake, 1993; Cabrera et al., 2019).

The arbitrary perspective presents the following strengths: (1) it separated the study of behavior from questionable explanations in terms of “mental life” or animal instincts; (2) it focused on the development of general theories; (3) it encouraged the development of standardized experimental tasks and paradigms allowing the comparison of general processes in different species—including humans (Timberlake, 1984); and (4) it encouraged the development of a parametric system for the study of behavioral processes (Schoenfeld et al., 1972).

On the other side, the ecological perspective emphasizes the following: (1) the functional differences of the experimental space and their effects on the behavior patterns, for example, the *forward-going tendency*, the *centrifugal swing*, the *direction of food turn* (Maier and Schneirla, 1964); (2) the role of locomotor activity on changes in the contact stimulation; (3) animals responding to invariants as well to changes in stimulation, that is, to the permanent properties of the environment as well as to their own motions; and (4) animals' capacities not only of locomotion in but also of orientation to their environment (Gibson, 1979).

In a parametric system, derived from an arbitrary approach, a functional relation between parametrical variations in stimuli properties and parametrical variations in behavioral patterns is assumed (Schoenfeld et al., 1972; Cabrer et al., 1975; Hernández and Ruiz, 2019; Hernández et al., 2020). Among the most prolific systems for the parametrical analysis of behavior are reinforcement schedules (Ferster and Skinner, 1957). In a standard reinforcement schedule, the occurrence of a relevant stimuli, for example, delivery of a food pellet or a drop of water, is contingent (i.e., it is dependent) on a discrete response of the organism (e.g., pressing a lever); these schedules are called contingent schedules. However, there are other stimuli schedules that do not require a response (or responses) in order to present relevant stimuli (e.g., delivery of drop of water), and they only depend on the temporal relations between stimuli, namely, non-contingent schedules (Zeiler, 1968; Lachter et al., 1971). The non-contingent schedules research is relevant, among other reasons, because, in the effort to understand the dynamics of the functional relations between behavioral patterns *not prescribed*, and *programed contingencies*, behavior analysis was extended beyond the analysis of discrete responses, adding the spatial dimension of the continuum of behavior (Pear and Eldridge, 1984; Pear, 1985, 2004).

In this way, the analysis of the spatial dynamics of the behavioral continuum under contingent (Pear, 1985) and non-contingent schedules (Pear and Eldridge, 1984; Silva and Pear, 1995; Silva and Timberlake, 1998; Hurtado-Parrado et al., 2019) and its integration with discrete-responses patterns, like *a whole system*, has become a relevant field for the analysis of behavioral processes. This emerging experimental field has gained strength with the development of new technologies, for example tracking systems (Pérez-Escudero et al., 2014) and tools for the analysis of data based on displacement patterns (León et al., 2020). An *integrated system of discrete responses* (e.g., lever pressing, nose poking, head inputs) *and displacement patterns* (e.g., routes) bring the arbitrary approach closer to the ecological approach since it allows, from the arbitrary approach, to characterize and analyze behavioral patterns with ecological relevance (e.g., water-seeking behavior) but in a parametric perspective (Hernández et al., 2020). Nevertheless, beyond the incorporation of behavioral patterns with ecological relevance in behavior analysis, the consideration of facts and evidence of the ecological approach can be useful for expanding the comprehension of the dynamics of the behavioral processes in a parametric way (Timberlake, 1993; Cabrera et al., 2019). One of the well-known facts of the ecological approach is the differentiation in the organization and dynamics of displacement patterns of rats (e.g., routes, distance, velocity, time spent) concerning different zones in the environment and even in environments in which the boundaries to the displacement of rats are a remote (e.g., a parking lot or a playing field) in comparison with standard open-field arenas (Whishaw et al., 2006).

In a work by Yaski et al. (2011), the dynamic of displacement patterns was studied under two forms of an open-field test (i.e., square, 200 × 200 cm, and round, 200 cm diameter). Sixteen rats were exposed to a single 20-min trial in each arena. The displacement of organisms was recorded with a two-dimensional tracking system. Routes, time spent per zone, distance, and velocity were analyzed concerning two virtual zones: peripheral and central. Results showed a robust difference in all referred measures in the two zones, independently of the shape of the arena. On the one side, rats showed a clear preference in terms of routes, time spent in zone, and distance traveled to the peripheral zones. On the other hand, velocity in the central zone was twice as high as in the peripheral zone. These findings show a functional difference between zones regarding the dynamics of displacement patterns.

In another work by Martinez and Morato (2004), the preference of rats to spend time in different zones in a modified open-field system that allowed to vary the number of close and open spaces was studied. Two brick blocks (20 × 20 cm of base) were used to create three-wall circumscribed zones (10 × 10 cm) in a squared experimental arena of 1.20 × 1.20 m. The arena was segmented according to the number of surroundings walls (0, 1, 2, 3, 4). One group of rats was exposed to 25, 10-min sessions. In the first 10 sessions, rats spent more time in the zones with two surrounding walls (i.e., the corners), while in the last 13 sessions, they spent more time in the zones with four walls. Time spent in zones without walls (i.e., central area) was near to zero in all sessions. In the two previously referred studies (Martinez and Morato, 2004; Yaski et al., 2011), the authors discussed their results from an ecological approach, in terms of the ecological relevance of each zone as the close zones serving as a refuge or “safe area” while characterizing the central zone as an “insecure area” for organisms (Whishaw et al., 2006).

As it was already mentioned, in a parametric approach, a functional relation between variations in stimuli properties and variations in behavioral patterns is assumed (Hernández and Ruiz, 2019; Hernández et al., 2020). Regardless of programmed contingencies, this approach generally assumes an undifferentiated functional space of the experimental arena. However, the findings of studies as those previously mentioned have important implications insofar as they show a clear functional differentiation in the dynamic of displacement patterns concerning different zones of the experimental arena even without programed contingencies.

Continuing with the parametric approach, time-based schedules are the most used non-contingent schedules. In a fixed time (FT) schedule, a relevant stimulus (e.g., drop of water) occurs at regular intervals (e.g., every 30 s) regardless of the activity of the organism, while in a variable time (VT) schedule, a relevant stimulus occurs at different intervals (e.g., x, y, z) whose mean is a given value (e.g., 30 s). In the standard arbitrary approach, the main data in the time-based schedules has been based on discrete responses, for example, dispenser entrances or, in some cases, the rate of response to a non-functional operandum (Skinner, 1938). But with the developments of Pear and Eldridge (1984); Hurtado-Parrado et al. (2019), relevant data based on displacement patterns have been added, for example, routes of displacement and distance between the organism and relevant objects (e.g., distance of organism to water/food dispenser).

A robust finding concerning the spatial dimension of behavior in time-based schedule is that, in fixed-time schedules, the routes are extended and unpredictable but consistent in an idiosyncratic way (Eldridge et al., 1988). On the other hand, the distance of organisms to the dispenser is usually greater in fixed-time schedules than in variable-time schedules (Van Hest et al., 1986). Nevertheless, to our knowledge, there are no studies that compare the spatial–temporal dynamics under FT vs. VT schedules.

There are some relevant issues about the parametrical approach that deserve to be mentioned: (a) the explanation of the spatial dynamics of behavior is based only on the programed contingencies; (b) the relevant objects (e.g., operanda and dispensers) and events (e.g., delivery of water or food) are concentrated on the peripherical zone (e.g., walls) of the experimental arena; and (c) an undifferentiated space is assumed in an ecological sense (i.e., the different zones of the experimental arena are assumed to be equifunctional).

From an integrative, parametrical–ecological approach, it is plausible to suppose that the intersection of the programed contingencies (e.g., time-based schedules), the ecological-differentiated space (e.g., peripherical vs. center zone), and the relative location of the relevant objects and events (e.g., location of the water source—peripherical vs. center zone) conform a system that is integrated with the behavioral patterns of the organism. Under this rationale, different ecologically relevant locations of a water source would set up different spatial dynamics of behavior under the same-programmed contingencies. In order to test this hypothesis, in the present study, we evaluated the relevance of two ecological locations of the dispenser upon the spatial dynamics of behavior in Wistar rats under two temporally scheduled deliveries (fixed- and variable-time schedules) in a modified open-field system.

## Experiment 1

### Method

#### Subjects

Three experimentally naive, female Wistar rats were used. All rats were 3 months old at the beginning of the experiment. Rats were housed individually with a 12-h light and dark cycle and maintained under a daily schedule of 23 h of water deprivation with free access to water 1 h after experimental sessions. Food was freely available in their home cages. One session was conducted daily, 7 days a week. All procedures were conducted in agreement with university regulations of animal use and care and followed the official Mexican norm NOM-062-ZOO-1999 for Technical Specification for Production, Use and Care of Laboratory Animals.

#### Apparatus

**Figure 1:**
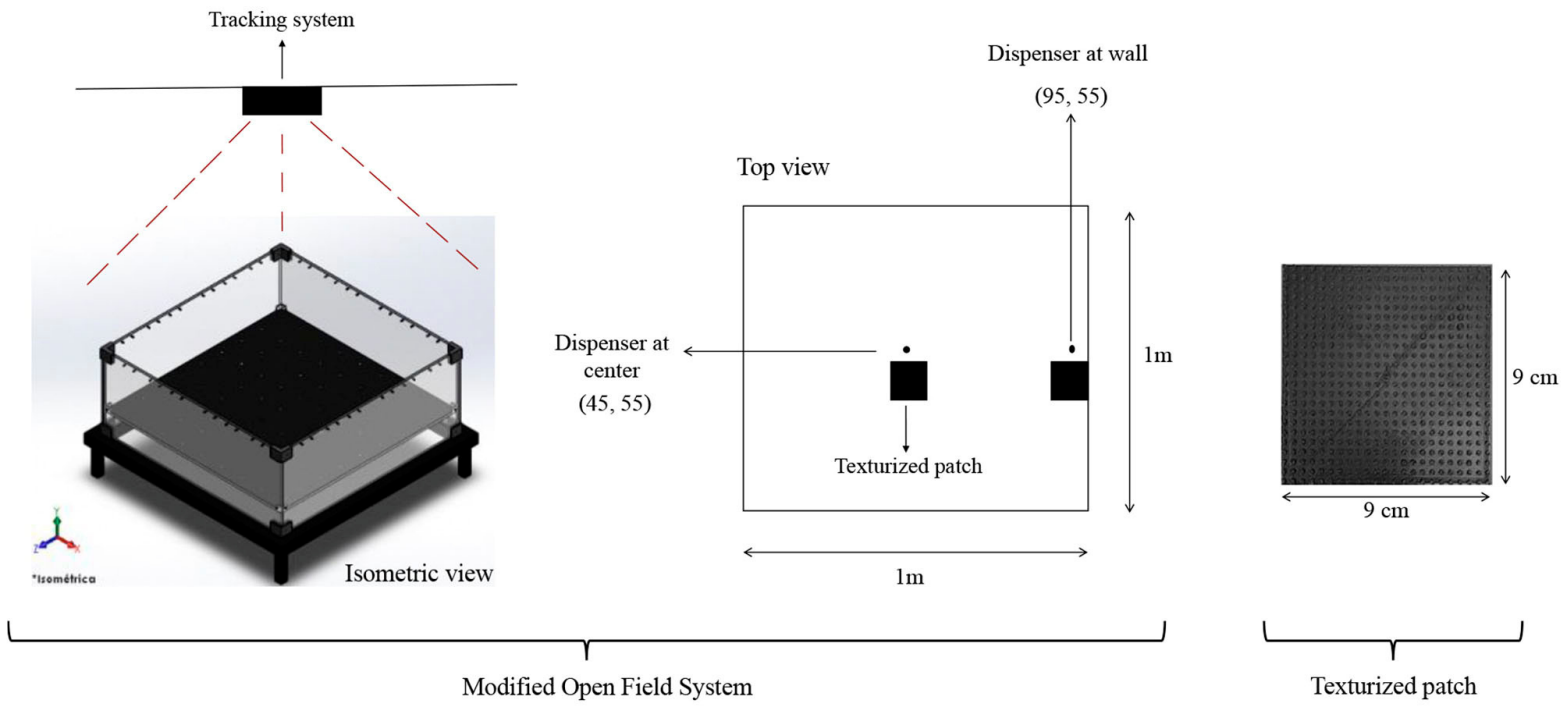
Diagram of the Modified Open Field System (MOFS). An isometric and top view of the system and its devices are presented on the left size of the figure. An image of the texturized patch is presented on the right side of the figure.

A modified open-field system (MOFS) was used. Figure 1 shows a diagram of the apparatus. Dimensions of the chamber were 100 × 100 cm. All four walls of the chamber as well as the floor were made of black Plexiglas panels. The floor contained 100 holes of 0.8 cm located 0.95 cm from each other. A water dispenser, based on a servo system, made by Walden Modular Equipment, was located close to the center of the (MOFS) (Condition I) or close to a wall (Condition II). When activated, it delivered 0.1 cc of water on a water cup that protruded 0.8 cm from the floor of the MOFS in one of the holes. The MOFS was illuminated by two low-intensity lights (3 W) located above the chamber and in opposite sides of the room in order to avoid shadow zones. Once delivered, water remained available 3 s for its consumption. A texturized black patch, 9 × 9 cm with 16 dots/cm, printed in a 3D printer, was located in close proximity (5.5 cm) to the water dispenser in order to facilitate its location.

The experimental chamber was located on an isolated room on top of a table of 45 cm of height. The room served to isolate external noise. All programmed events were scheduled and recorded using Walden 1.0 software. The rats' movement was recorded by a Logitech C920 web camera, located at the center, 1.80 m above the experimental chamber. Tracking data were analyzed using Walden 1.0 software. This software recorded the rats' location every 0.2 s in the experimental space using a system of X, Y coordinate. The system recorded the rats according to their center of mass. Data files obtained from this software were then analyzed using MOTUS™ and SPATIUM software.

#### Procedure

Subjects were exposed to two consecutive conditions in the same order (See Table 1). On each condition, water was delivered using an FT 30-s schedule. When delivered, water remained available for 3 s. In Condition I, the water dispenser was located on the floor at the center of the experimental chamber (see Figure 1). In Condition II, the water dispenser was located on the floor next to a wall. Each condition lasted 20 sessions. Each session lasted 20 min. Rats were directly exposed to the conditions without any previous training. The MOFS was cleaned using isopropyl alcohol between each experimental session.

**Table 1:** Experimental design of experiments 1 and 2.

**Experiment**	**Water-delivery schedule**	**Condition I**	**Condition II**
1	Fixed time	Center	Wall
2	Variable time	Center	Wall

### Results

**Figure 2:**
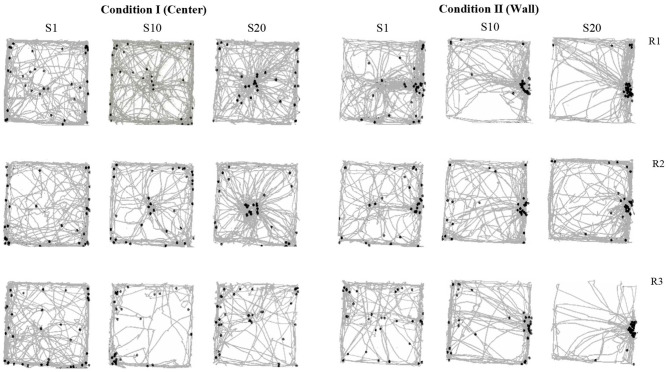
Route per session. Each panel shows the analogic routes in the MOFS for a complete session. Black points show rats' location in the arena at the first moment of water delivery (the first frame of 0.2 s of the 3 s of water availability). Each row depicts data for one rat, and each column depicts routes for sessions 1, 10, and 20 for conditions I and II of Experiment 1.

Figure 2 shows rats' displacement on the MOFS and rats' location in the arena every 0.2 s for the duration of the entire session. The rats' location in the first 0.2 s of water delivery is indicated with black marks. It is important to mention that water remained available for 3 s each time, so even if the rats' location at the beginning of the interval was not close to the dispenser, they still could approach and make contact with it. The first three columns depict data for sessions 1, 10, and 20 of Condition I (center), and the next three columns depict data for sessions 1, 10, and 20 of Condition II (wall). Data for rats 1, 2, and 3 are shown in separate rows. In Condition I, in the first sessions, all three rats moved predominantly close to the walls of the MOFS with some crossing between walls. In session 10, rats 1 and 2 continue to move predominately on the walls, but a pattern of visits to the center began to clearly appear. For rat 3, movement stayed close to the walls with very little crossing to the walls using the center of the floor. In session 20, for rats 1 and 2, there was a clear back and forth pattern to the center of the chamber and to the walls of the MOFS. For rat 3, there were some crossing in the middle of the MOFS compare to session 10 but without the clear pattern shown by rats 1 and 2.

In Condition I, in the first session, for all subjects, the rats' location at the moment of water delivery was far from the dispenser and close to the walls with only a few instances in which rats' location was close to the water dispenser at the moment of delivery. This pattern remained similar for session 10 with just a few instances in which the rats were located close to the dispensers; nonetheless, a clear differentiation in terms of displacement began to appear among the walls and the center of the arena. For session 20, the number of instances of water delivery with the rats close to the dispenser slightly increased, and a clear differentiation in the location of the rats between the center and walls was found.

In Condition II, in the first session, rats 1 and 2 showed a clear back and forth pattern of travels between the center of the floor to the wall where the water dispenser was located. This pattern was also shown in rat 3 but less clearly. In session 10, the movement of all three rats concentrated predominately on the wall with the water dispenser, although there were still some crossings in the middle of the floor. In session 20, all three rats predominately stayed close to water dispenser's wall with less crossing by the middle of the floor in comparison to session 10.

In Condition II, for all subjects from session 1, there was a drastic change in the rats' location at the moment of water delivery with more instances in which they were close to the dispenser's location in comparison to the previous condition. In session 10, the number of times in which rats were in close proximity to the dispenser at the moment of water delivery clearly increased with just a few instances in which these did not coincide. This pattern was maintained and highlighted in session 20 for all rats. In short, in Condition I rats' location in the first 0.2 s of water delivery was distributed between the perimetral and central zone (close to dispenser), while in Condition II, it concentrated in the perimeter and dispenser zone.

**Figure 3:**
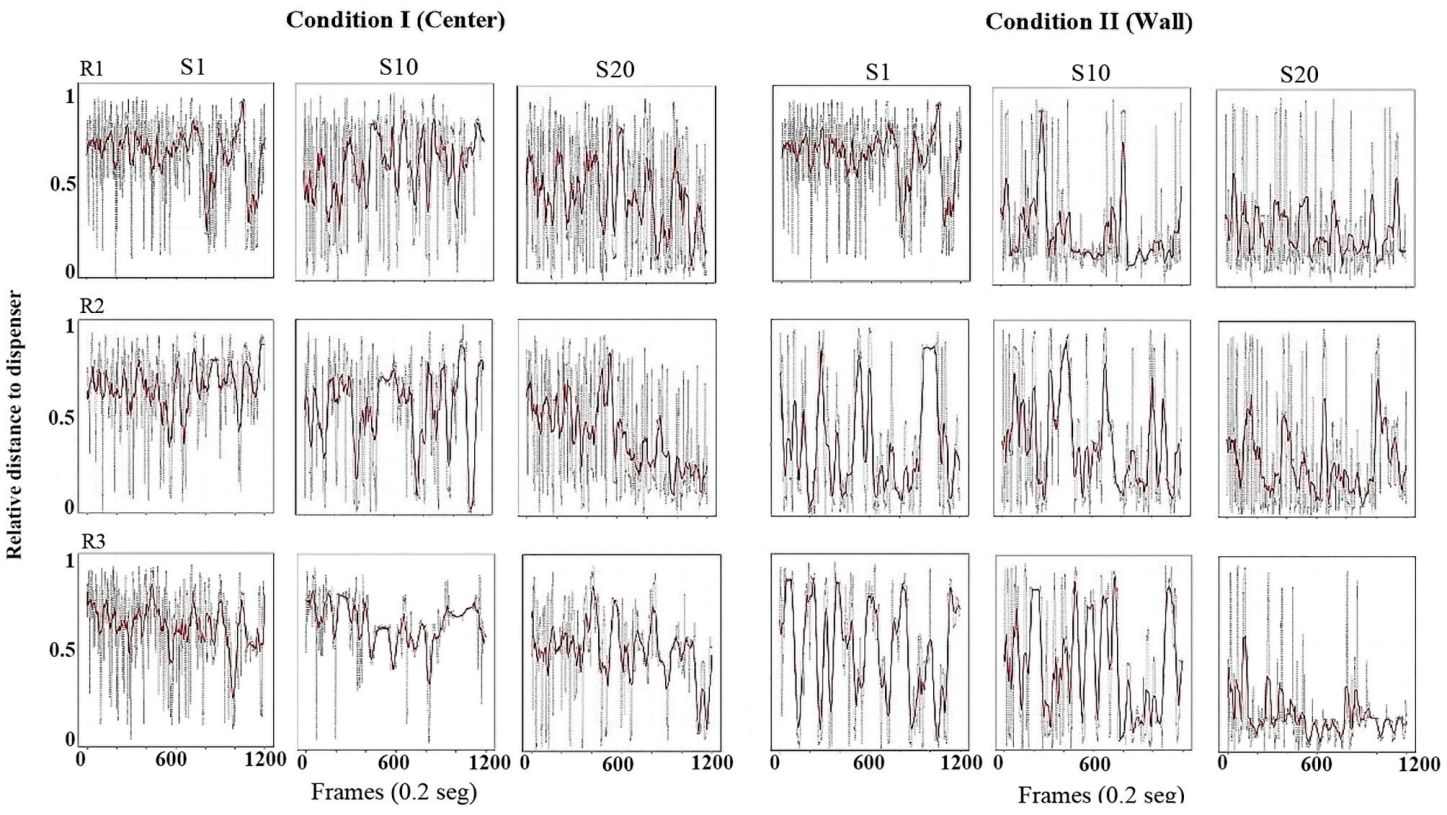
Distance to dispenser. Each panel shows the relative value of the distance (0 = minimum to 1 = maximum) from the rat to the dispenser, every frame or 0.2 s (gray dots) and a moving average of 200 frames (red line) for a complete session. Each row depicts data for one rat, and each column depicts data for sessions 1, 10, and 20 for conditions I and II of Experiment 1.

Using the same format as the previous figure, Figure 3 shows the relative value of the distance from the rat to the dispenser every 0.2 s (gray dots). To obtain these values, the maximum possible distance from the rat to each location of the dispenser (center or wall) was calculated, and the distance every 0.2 s was divided between the maximum distance corresponding to that condition. In addition, in order to clearly show the tendency of the distance function, we performed a smoothing of it (red line) by using a moving average of 200 frames (i.e., 40 s, see equation in Appendix A). With this measure, a value close to 1 would indicate that the distance from the rat to the dispenser was the maximum possible for that location of the water dispenser. A value close to zero would indicate that the rat was located in close location in relation to the dispenser. If the only relevant object were the water dispenser, it would be expected that this distance decreased as sessions continued, independently of the location of the dispenser (wall or center).

In Condition I, in the first session, the three rats' distance from the dispenser was elevated, with most values close to one and minor variation along the sessions. In session 10, also for all subjects, relative-distance values continue to be close to the maximum possible, but a pattern of increases and decreases in the distance began to appear varying from almost 1 to values close to 0. In session 20, the values showed a clear pattern to increase and decrease but with distance close to the lower values, mainly at the end of the session for all rats.

In Condition II, with the dispenser located in a wall of the chamber, from session 1 and through all sessions, there was a clear pattern for the value of the distance to remain close to the lower values, although with some variability across sessions.

**Figure 4:**
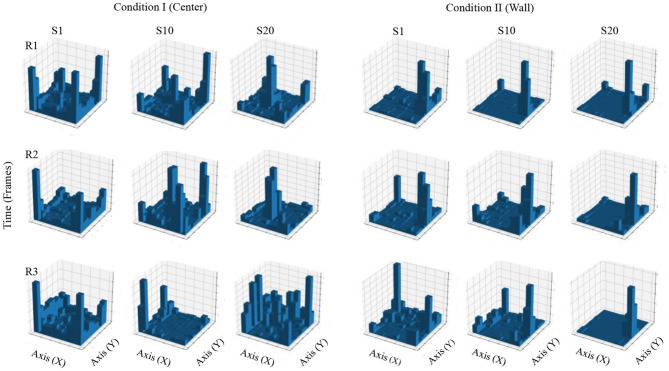
Accumulated time of stays. Each panel shows the accumulated time of stays in a square region from a configuration of 10 × 10 zones. Each row depicts data for one rat, and each column depicts data for sessions 1, 10, and 20 for conditions I and II for Experiment 1.

Figure 4 shows accumulated time of stays in each square region from a configuration of 10 × 10 defined zones. In Condition I, in the first session, accumulated time for all three rats was higher in the regions close to the walls and on the corners of the MOFS. In session 10, for rats 1 and 3, stays had longer accumulated time close the walls, although there was an increase in time spent at the center of the experimental chamber, whereas rat 3 showed longer stays close to one wall of the chamber. In session 20, accumulated time of stays was higher in the central regions of the chamber for rats 1 and 2, compared to accumulated time close to the walls. For rat 3, accumulated time was higher for the regions in close proximity to the walls, but it also increased in the central areas of the chamber.

In Condition II, in the first session, accumulated time for all three rats was higher in the region close to the wall where the water dispenser was located with minor stays at the center of the chamber. For sessions 10 and 20, stays were longer and concentrated in zones close to the wall of the water dispenser.

**Figure 5:**
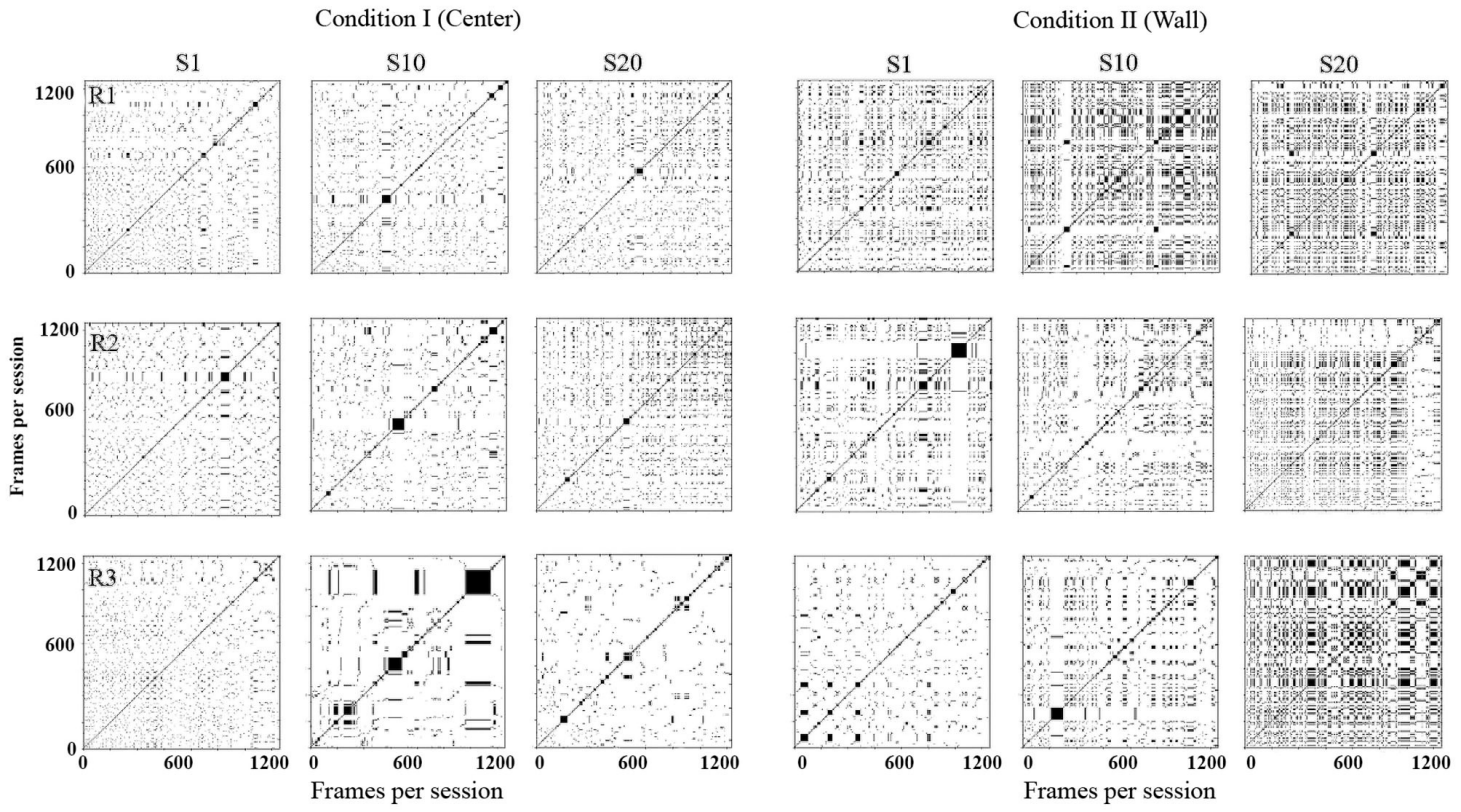
Recurrence plots. Each panel depicts change of regions for each rat in a configuration of 10 × 10 defined zones every 0.2 s. Each row depicts data for one rat, and each column depicts data for sessions 1, 10, and 20 for conditions I and II for Experiment 1.

Figure 5 shows recurrence plots. This plot depicts change in regions of each rat in a configuration of 10 × 10 defined zones comparing the rats' location along time [for a complete description, see León et al. (2020)]. Both axes show time on a time frame of 0.2 s. If a rat was on an *R*_*k*_ region in a T time and on T + 1 on the same region, a black mark represented the recurrence in time. If, on the contrary, on T + 1 the rat was on a different location, a white mark would be shown. The recurrence plot shows the reiteration of the organism's location in a given value in time (frame per frame). Appendix B shows the equation employed to obtain the recurrence plots.

With this analysis, it is possible to identify the return and permanence of the organism to a region. The densification and alternation of black–white mosaic patterns indicates high recurrence; a higher proportion of continue black zones would mean higher permanence in a given region, while a higher proportion of white zones would mean higher transitions among regions.

In Condition I (center), in the first session, there were high transitions among regions along time (densification of white). In session 10, rats 1 and 2 continue to show high transitions but with low recurrence (densification and alternation of black–white mosaic patterns). For rat 3, the level of recurrence increased in comparison to session 1. In session 20, for rats 1 and 2, recurrence slightly increased in comparison to sessions 1 and 10; also recurrence started to increase by the end of the sessions. For rat 3, recurrence level remained low.

In Condition II (wall), there were higher transitions among regions along time (densification of white). In session 10, recurrence increased for rats 1 and 3 and remained low for rat 2. In session 20, recurrence increased for all three rats. In general, recurrence was higher for all sessions in Condition II compared to sessions in Condition I.

With the purpose of having a quantitative measure of the variation of displacement patterns, an entropy analysis was conducted. This analysis provides a quantitative index of the variations of displacement patterns within sessions, with high entropy representing more variation of displacement patterns and low entropy representing less variation of displacement patterns. The mathematical description of this analysis can be found in Appendix C.

**Figure 6:**
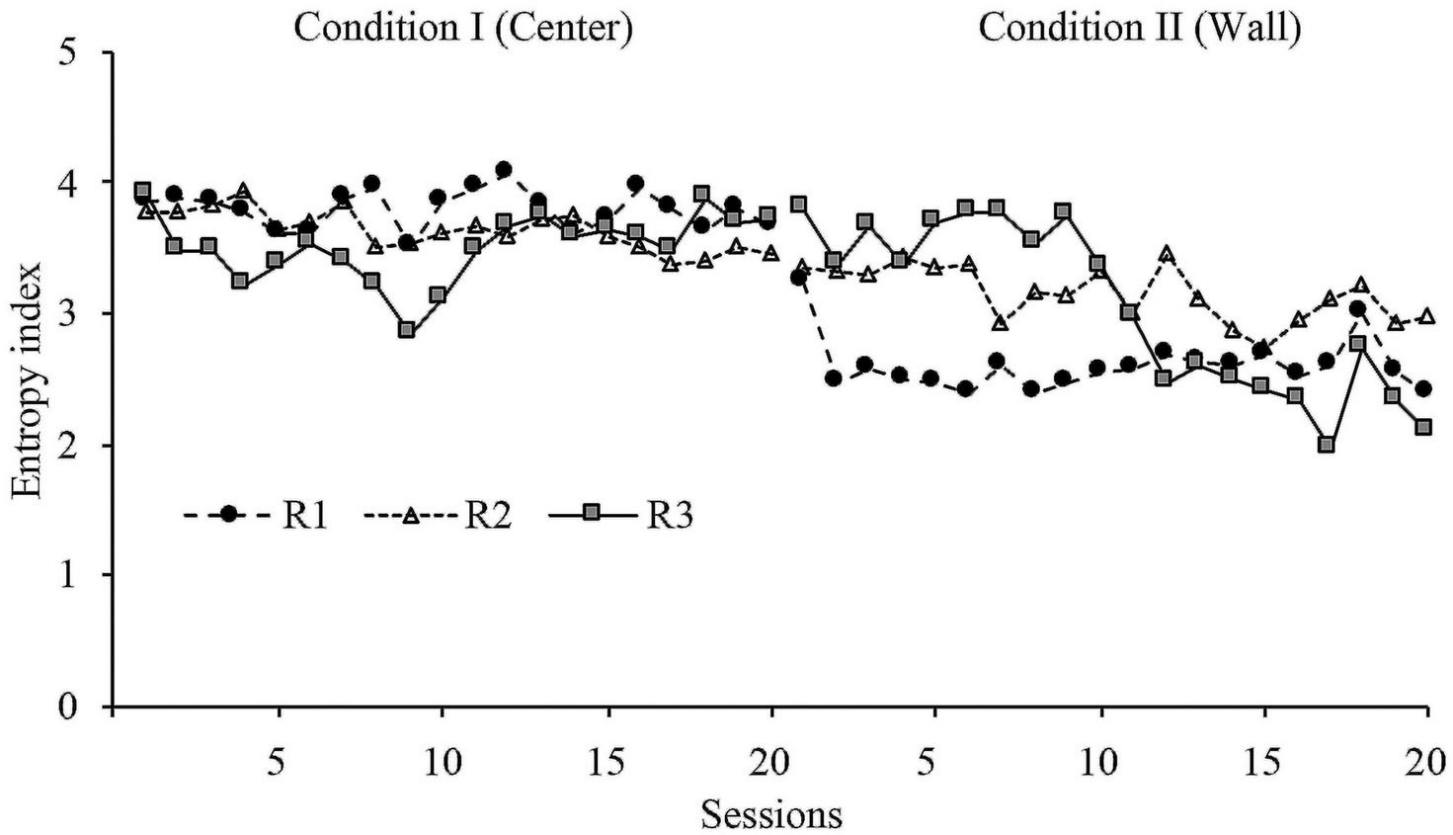
Entropy index. Entropy values for each rat across all sessions of Condition I and Condition II for Experiment 1.

Figure 6 shows the entropy values for all sessions in conditions I and II. The entropy index in Condition I showed a very stable level for the second part of the phase. In Condition I, it showed an abrupt change in level for R1 and a delayed change in level for R2 and R3. The change in level for R3 was very robust. The entropy index in the last sessions were markedly different between conditions but consistently within conditions.

Entropy did not show a major change within phases, but it showed a significant change between phases. These findings shows (a) that the behavioral dynamics within phases was consistent, and (b) there was a wide difference in behavioral dynamics between both dispenser's location: when the dispenser was allocated at the center zone, the displacement patterns showed more variation than when the location was in the perimeter zone.

With the purpose of determining the variations in displacement patterns between sessions, a divergence index was calculated considering all sessions. This index is calculated by comparing the displacement patterns between two consecutive full sessions (e.g., 1 and 2; 2 and 3, etc.). A value close to 0 indicates no difference in the displacement patterns between full sessions; a positive value indicates difference in displacement patterns between full sessions. The mathematical description of this analysis can be found in Appendix D. The expected KL divergence were values near to 0 between sessions of the same phase and high values between sessions of the phase transition (change in the dispenser location).

**Figure 7:**
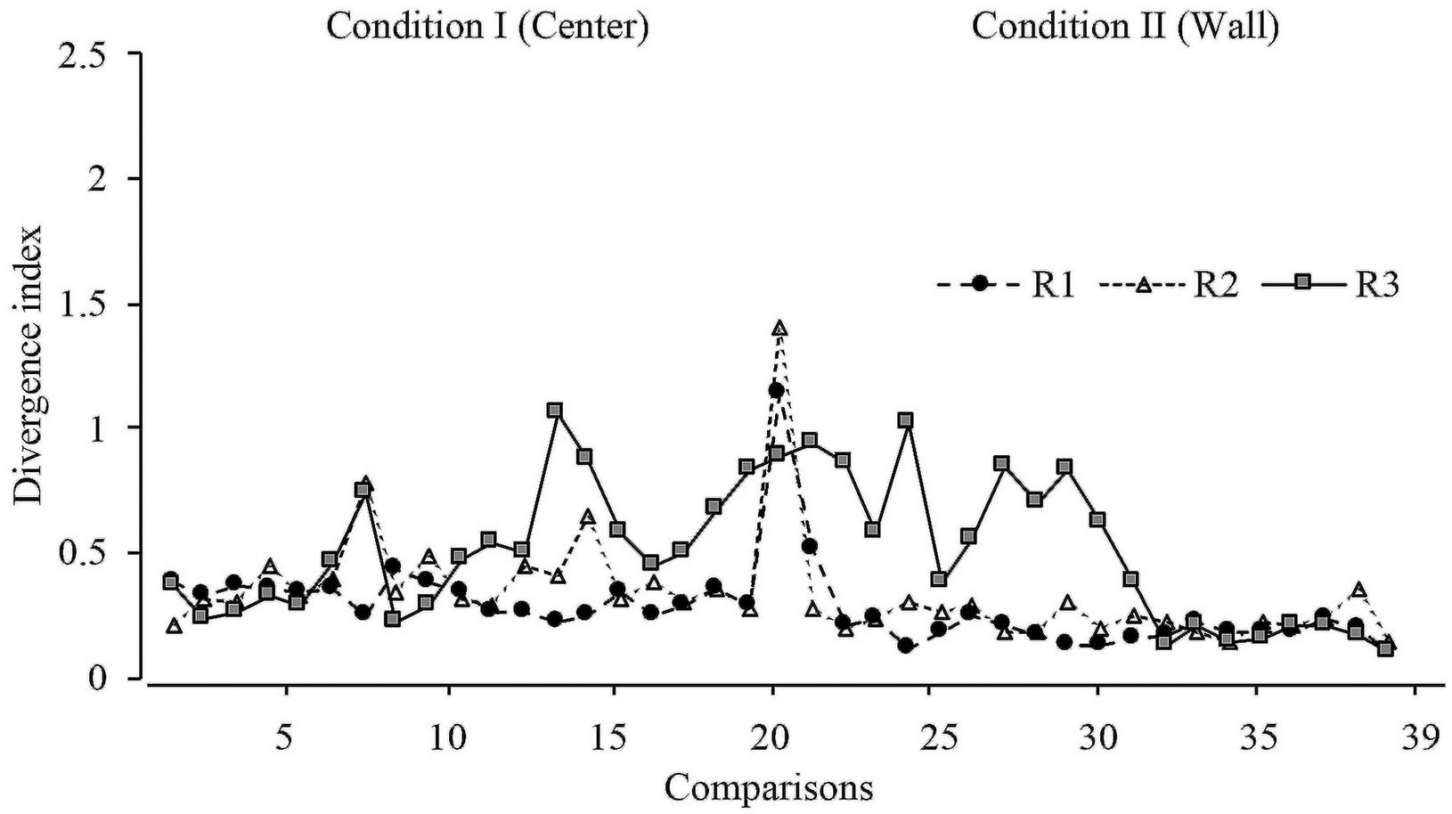
Divergence index. Divergence values for each rat across all sessions of Condition I and Condition II for Experiment l.

Figure 7 depicts the divergence analysis throughout all sessions in conditions I and II. For rats 1 and 2, the values remained close to 0, except in the first session from Condition II, in which the divergence value notably increased, showing a difference in displacement patterns when the location of the water dispenser was changed in consecutive sessions. For rat 3, the divergence index showed variations in the last 10 sessions of Condition I and the first 10 sessions in Condition II, but values remained close to 0 in the last eight comparisons.

## Experiment 2

### Method

#### Subjects

Three experimentally naive Wistar rats were used: two males and one female (subject 4). All rats were 3 months old at the beginning of the experiment. Housing conditions and maintenance were identical to the ones used in Experiment 1. All procedures were conducted in agreement with university regulations of animal use and care and followed the official Mexican norm NOM-062-ZOO-1999 for Technical Specification for Production, Use, and Care of Laboratory Animals.

### Results

**Figure 8:**
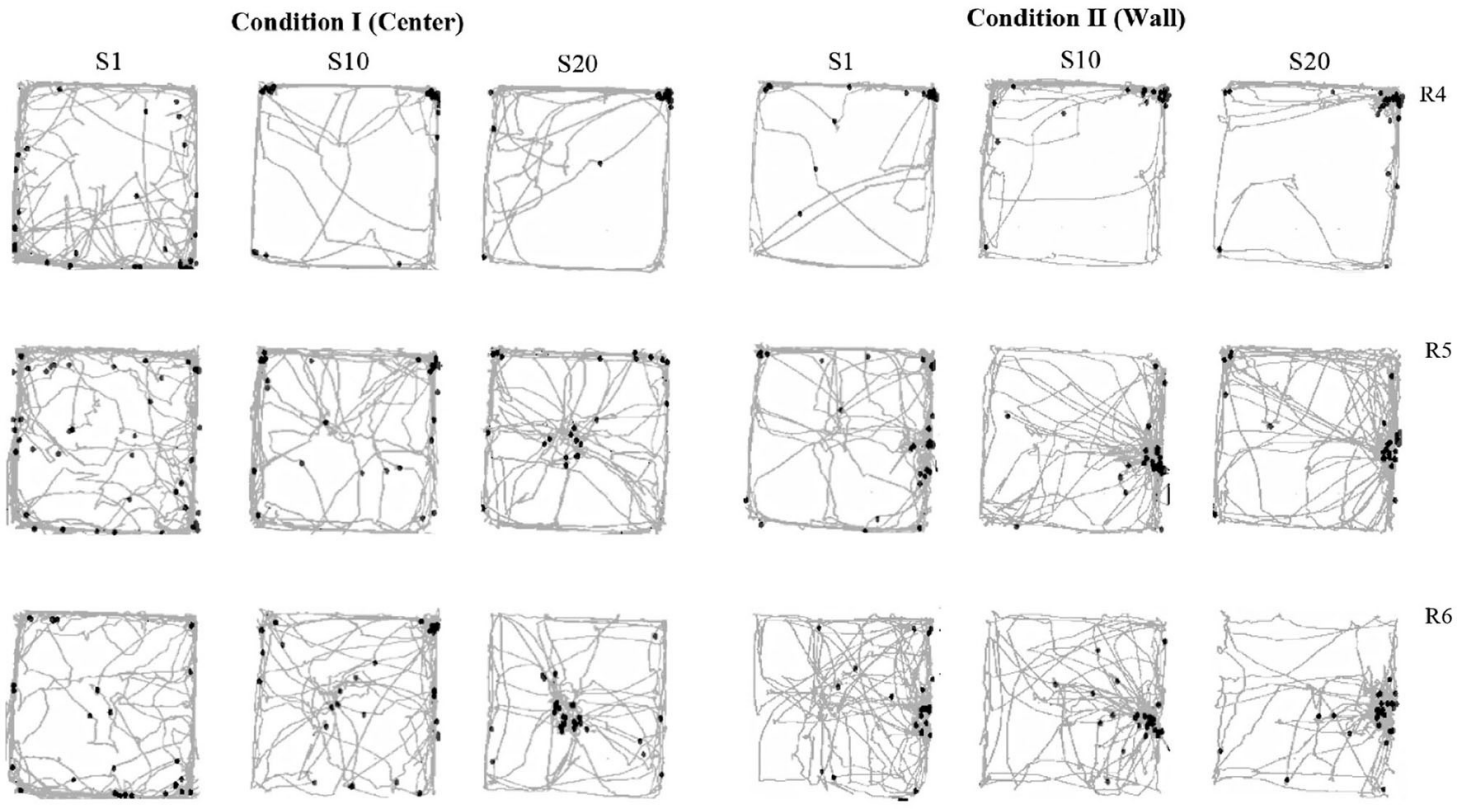
Route per session. Each panel shows the analogic routes in the MOFS for a complete session. Black points show rats ' location in the arena at the first moment of water delivery (the first frame of 0.2 s of the 3 s of water availability). Each row depicts data for one rat, and each column depicts routes for sessions 1, 10, and 20 for conditions I and II of Experiment 2.

The format for all figures in Experiment 2 is the same used in Experiment 1. Figure 8 shows rats' displacement on the MOFS and rats' location in the arena every 0.2 s for the duration of the entire session. The rats' location in the first 0.2 s of water delivery is indicated with black marks. In Condition I, in the first sessions, all three rats moved mostly close to the walls of the chamber with minor crossings between walls. In session 10, rats 5 and 6 predominately move close to the walls, but a pattern of visits to the center began to appear. For rat 4, routes continue to be close to the walls, and movement diminished in comparison to session 1. In session 20, for rats 5 and 6, the back and forth pattern to the center of the chamber and the walls of the MOFS was clearly present, whereas for rat 4, routes remained close to the wall.

In Condition I, in the first session, the location of all rats at the moment of water delivery was distant from the dispenser and close to the walls. There were only a few instances for rats 5 and 6 in which their location was close to the water dispenser at the moment of delivery. This pattern remained similar for session 10 with just a few instances in which rats were located close to the dispensers; nonetheless, a clear differentiation in terms of displacement began to appear among the walls and the center of the arena, especially for rats 5 and 6. In session 20, the number of instances of water delivery with the rats close to the dispenser increased for subjects 5 and 6, and a clear differentiation in the location of the rats between the center and walls was found also for those subjects. Rat 4 remained close to the walls at all times.

In Condition II, in session 1, rats 5 and 6 showed a back-and-forth pattern of travels between the center of the floor to the wall where the water dispenser was previously located. For rat 4, routes remained close to the walls of the arena. For sessions 10 and 20, the pattern of routes remained similar to previous sessions for all three rats with clear back-and-forth patterns for rats 5 and 6 and routes close to the walls for rat 4.

In Condition II, for subjects 5 and 6 from session 1, there was a clear change in location at the moment of water delivery with more instances in which they were close to the dispenser in comparison to the previous condition. In session 10, the number of times in which rats were in close proximity to the dispenser at the moment of water delivery clearly increased with just a few instances in which these did not coincide. This pattern was maintained and highlighted in session 20 for all rats. Rat 4 was not sensitive to the change in location of the water dispenser and, for all sessions, remained close to the walls.

**Figure 9:**
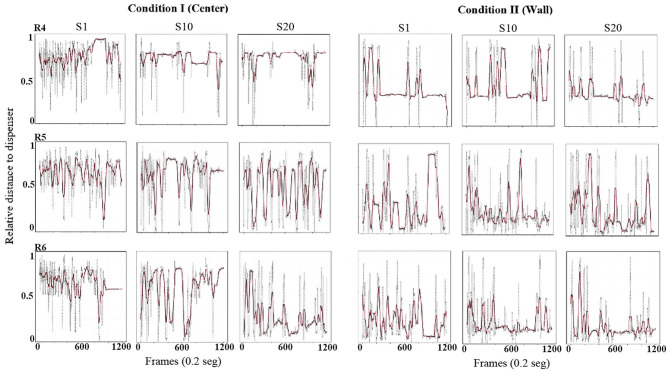
Distance to dispenser. Each panel shows the relative value of the distance (0 = minimum to 1 = maximum) from the rat to the dispenser, every frame or 0.2 s (gray dots) and a moving average of 200 frames (red line) for a complete session. Each row depicts data for one rat, and each column depicts data for sessions 1, 10, and 20 for conditions I and II of Experiment 2.

Figure 9 shows the relative value of the distance from the rat to the dispenser every 0.2 s (same formula described in Figure 3). In Condition I, in the first session for all rats, the distance to the dispenser was high and close the maximum values. For session 10, for rats 5 and 6, distance remained close to the maximum values but with variation to the lower values, while for rat 4, the pattern remained similar to session 1. For session 20, and for rats 5 and 6, distance values decreased, with some variability to high values. For rat 4, distance values had no clear change compared to previous sessions.

In Condition II, session 1, in general for all three rats, the distance to the dispenser was lower in comparison to Condition I. In sessions 10 and 20, the distance to the dispenser slightly decreased but with some fluctuating patterns toward intermediate values.

**Figure 10:**
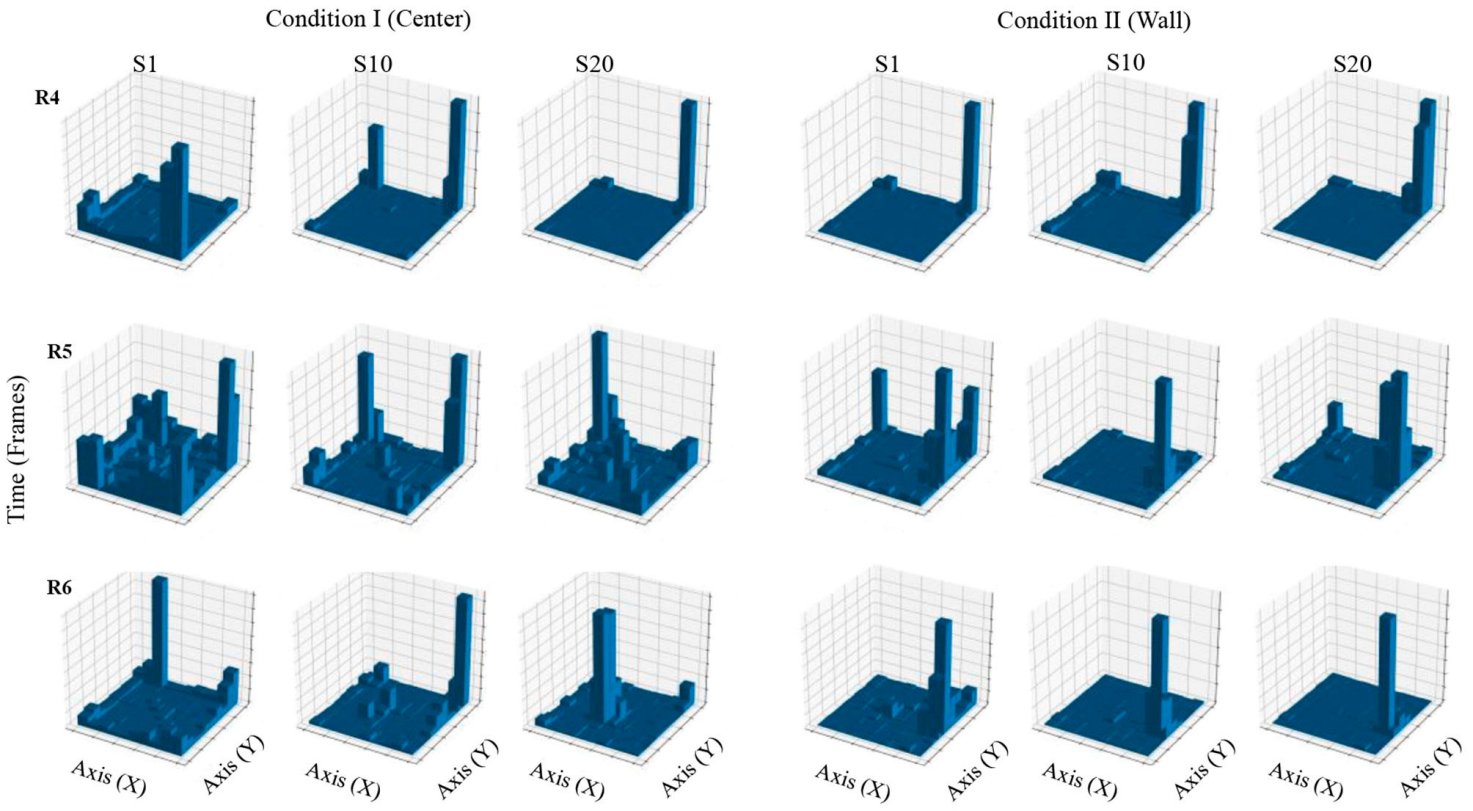
Accumulated time of stays. Each panel shows the accumulated time of stays in a square region from a configuration of 10 × 10 zones. Each row depicts data for one rat, and each column depicts data for sessions 1, 10, and 20 for conditions I and II for Experiment 2.

Figure 10 shows accumulated time of stays in each square region from a configuration of 10 × 10 defined zones. In Condition I, in the first session, accumulated time for all three rats was higher in the regions close to the walls and on the corners of the MOFS. In session 10, for rats 5 and 6, stays had longer accumulated time close to the walls, although time spent at the center of the experimental chamber increased. For rat 4, stays concentrated close to one wall of the chamber. In session 20, stays for rats 5 and 6 were longer in the central regions of the chamber, compared to previous sessions. Rat 4 remained to present longer stays close to one wall.

In Condition II, in the first session, accumulated time for all three rats was higher in the region close to the wall where the water dispenser was located; rats 5 and 6 also showed short stays at the center of the chamber. For sessions 10 and 20, for all three rats, stays were longer and concentrated in zones close to the wall of the water dispenser.

**Figure 11:**
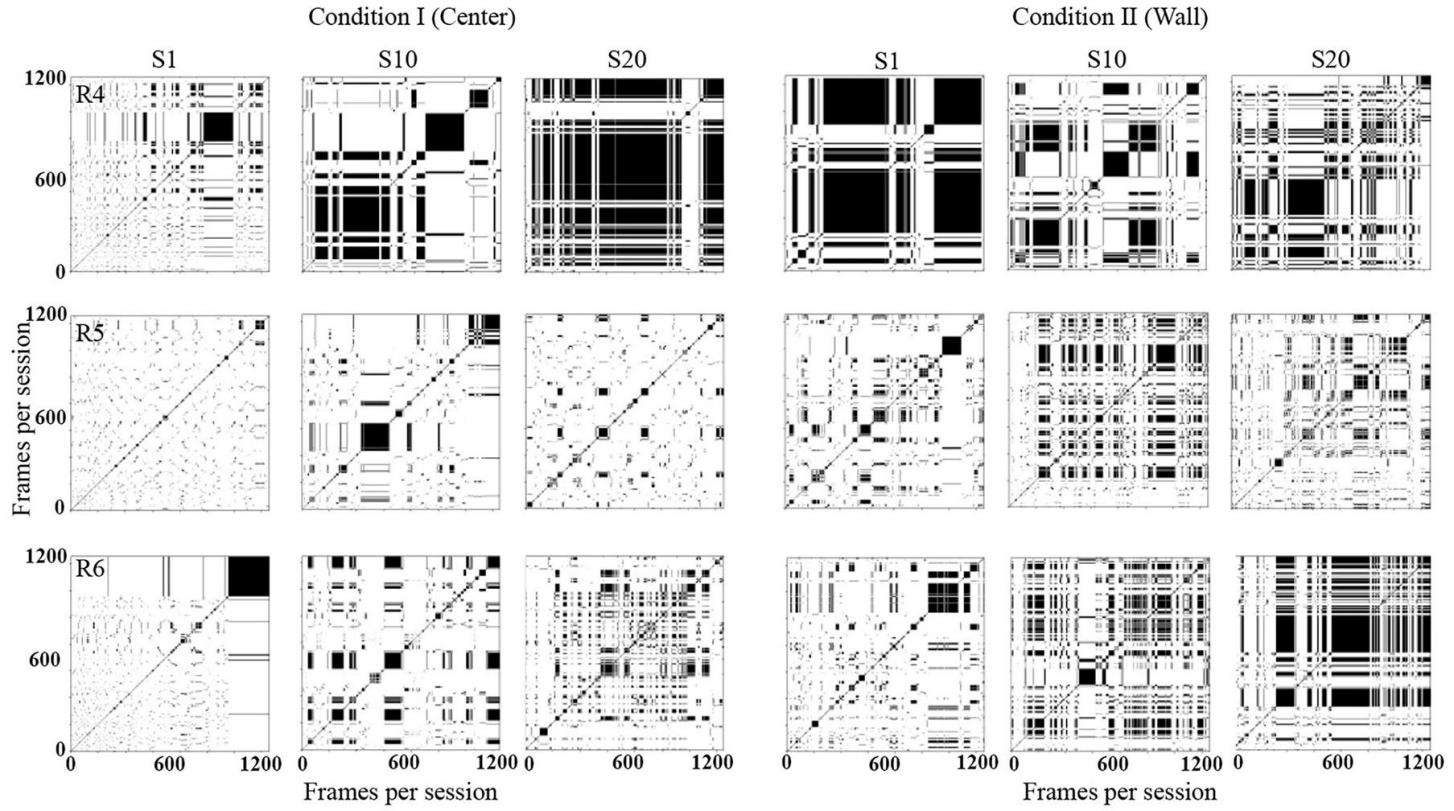
Recurrence plots. Each panel depicts change of regions for each rat in a configuration of 10 × 10 defined zones every 0.2 s. Each row depicts data for one rat, and each column depicts data for sessions 1, 10, and 20 for conditions I and II for Experiment 2.

Figure 11 shows recurrence plots. In Condition I (Center), in session 1, for all three rats, there were high transitions between zones with a decrement by the end of the session for rats 4 and 6. In session 10, rats 5 and 6 showed recurrence patterns compared to session 1, and rat 4 showed higher permanence in zones. In session 20, rat 4 showed higher permanence, while it remained similar to the previous session for rats 5 and 6.

In Condition II (wall), for rat 4, the permanence remained high and stable for session 1, while in sessions 10 and 20, some recurrence patterns emerged, indicating transitions among regions. For rats 5 and 6, in session 10, recurrence increased in comparison to previous sessions, and short stays increased in session 20. In general, the permanence in zones was higher for all sessions in Condition II compared to sessions in Condition I.

**Figure 12:**
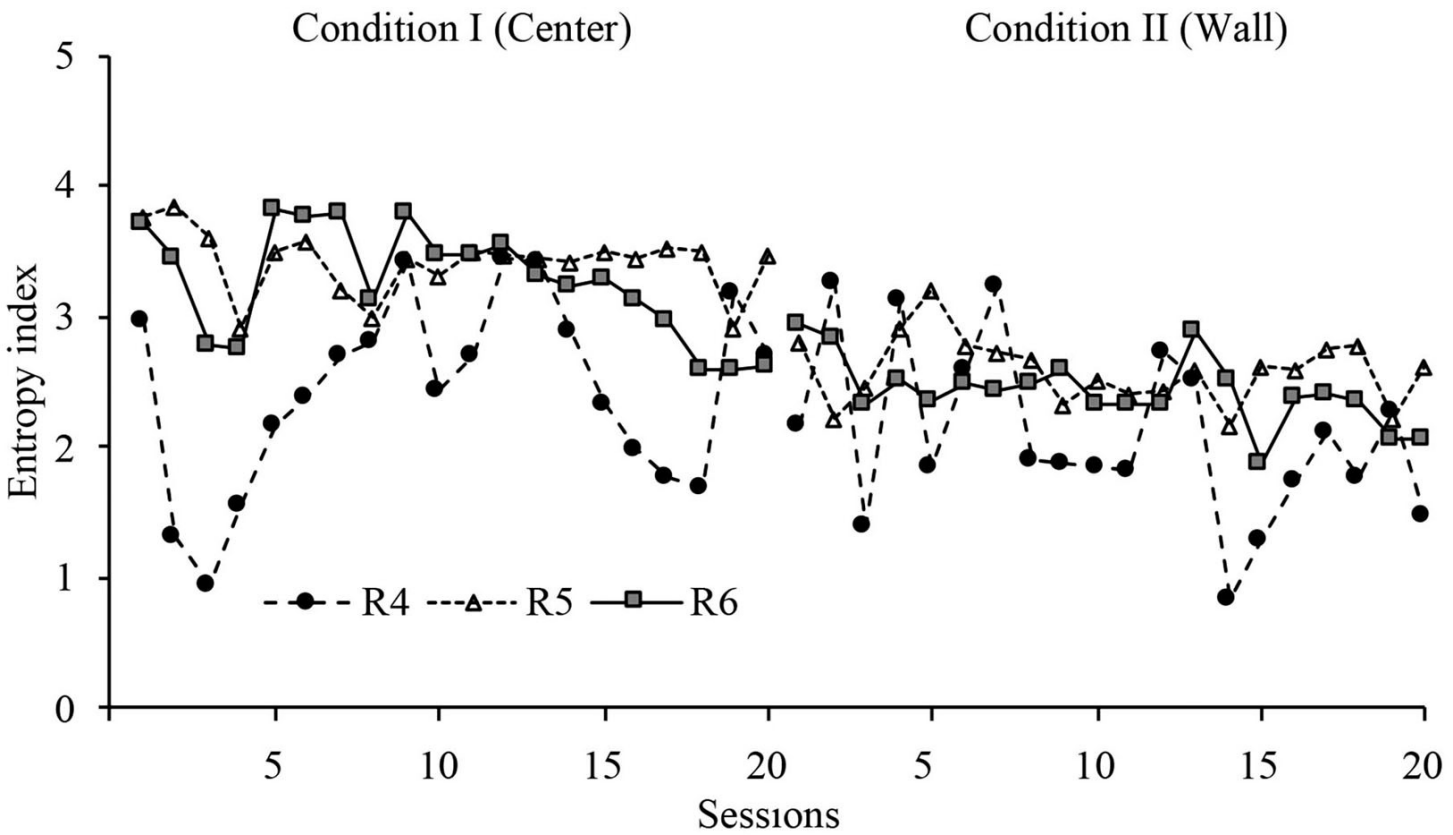
Entropy index. Entropy values for each rat across all sessions of Condition I and Condition II for Experiment 2.

With a similar format to Figures 6, 12 shows the entropy values for all sessions in Conditions I and II. The entropy index, in general, showed variability. However, in Condition I, it showed a stable level for the last eight sessions for R8 and the last three sessions for the R9. In Condition II, it showed an abrupt change in level for R8 and a delayed and smooth change in level for R9. The entropy index for the last sessions of Condition II was stable and lower than the last sessions of Condition I.

**Figure 13:**
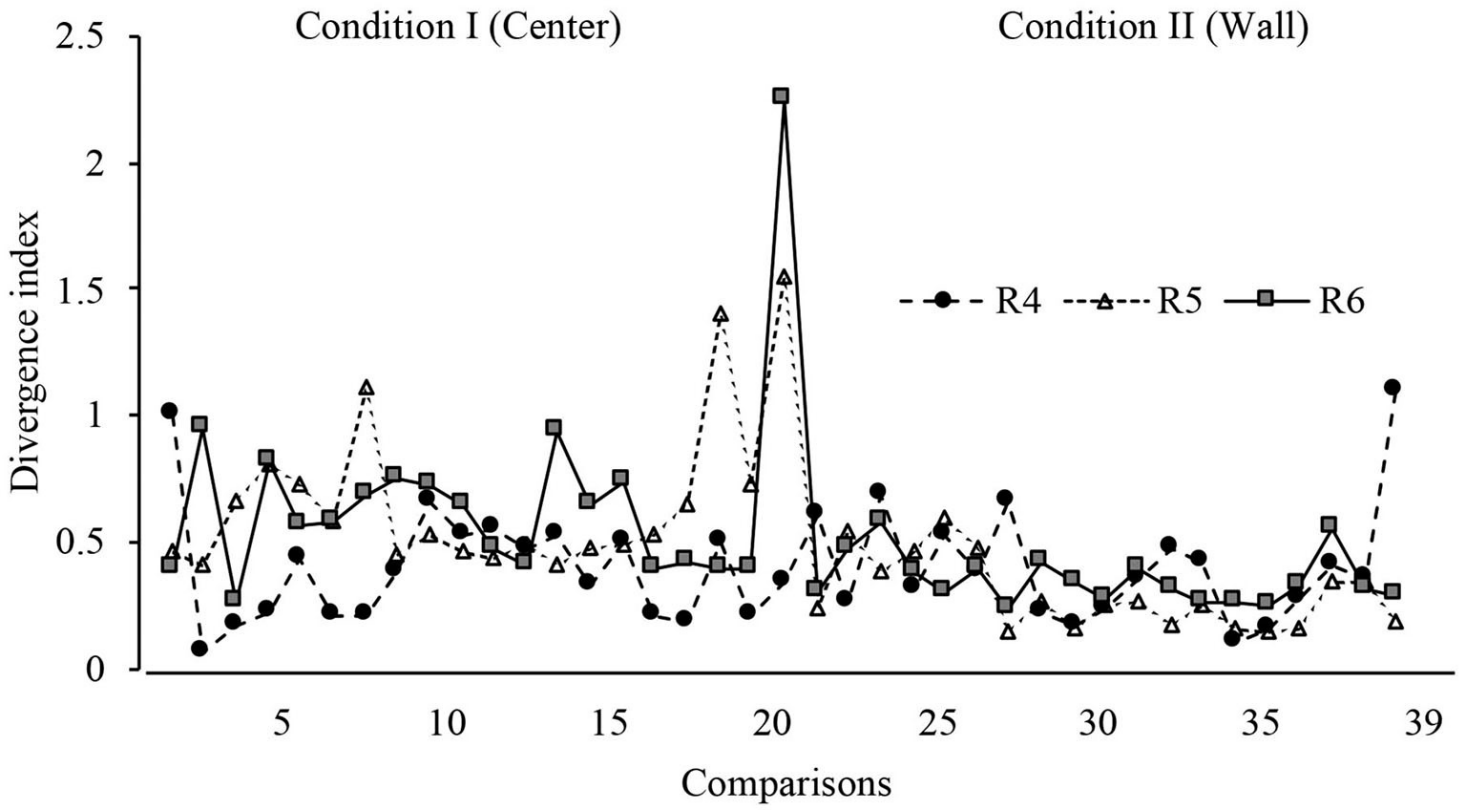
Divergence index. Divergence values for each rat across all sessions of Condition I and Condition II for Experiment 2.

With a similar format to Figures 7, 13 shows the divergence analysis for sessions in Conditions I and II. For rats 5 and 6, the values increased in the first session of Condition II, on the other hand, in general terms, the divergence value was lower in Condition II than in Condition I. For rat 4, there was no clear change in divergence values in the first session of Condition II.

## Discussion

The present study evaluated the relevance of two ecological locations of the dispenser upon the spatial dynamics of behavior in Wistar rats under two water-delivery schedules (fixed time and variable time) in a modified open-field system. In order to have an integrative and multidimensional characterization of the temporal–spatial dynamics of behavior, several analyses were conducted. In this section, we will briefly describe and discuss in an integrative and comparative manner the findings of both studies from an ecological-parametric framework.

In the first sessions of the initial phase, with water delivered at the center, for both studies, our results were consistent with the findings reported in the literature regarding displacement patterns in an open-field arena (Martinez and Morato, 2004; Whishaw et al., 2006; Yaski et al., 2011). That is, routes and time spent per zone concentrated in the perimeter of the arena, so a typical thigmotaxis pattern was observed, indicating an initial eco-functional segmentation of the experimental arena given the directionality and the preference of the patterns (approach patterns to the perimeter zone). Nevertheless, around session 10 of the first phase, a relevant change in the directionality of displacement patterns was observed, and these were oriented to both the center and perimeter zones. This analysis shows that, at the moment of water delivery, when the dispenser was at the center zone, the animals were usually far to the dispenser (in the perimeter zone), while when the dispenser was in the perimetral zone, the animals were near to the dispenser. The conjunction between the data of organism's location at the first moment of water delivery and the distance to the dispenser (see distance close to zero) suggests that, when the dispenser was at the center location, rats traveled from the perimeter to the center at the time of the water delivery. Thus, with the dispenser at the center, back-and-forth patterns associated with the deliveries take place, while with the dispenser in the perimeter, patterns of stays take place. This differentiated emergent patterns, with quantitative variations, can be observed under TF and TV schedules. This finding is important because the back-and-forth patterns between the central and perimeter zones displayed by the organisms indicate a new emergent eco-functional segment in the experimental arena: the center zone by the delivery of water. These patterns were very clear in the last sessions of the first phase. In consequence, by having two distant eco-functional segments (the dispenser and the perimeter zone) in the experimental area, the distance to the dispenser fluctuated between “close” and “far,” which implicated an underlying spatial dynamics with high recurrence of patterns, accelerated transitions between zones, and high entropy. These dynamics were more robust under the FT than under the VT schedules.

On the other hand, in the second phase, when the dispenser was located in the perimeter zone, a significant change in directionality was observed. That is, routes and time spent in zones primarily concentrated in the perimeter area, in the wall near to the dispenser with only some incursions to the center zone. Thus, the center area lost functional relevance, except for the change between phases, the back-and-forth patterns decreased, and the relevant or functional segments in the arena were contracted to the perimeter zone. On the other hand, the distance to the dispenser in the second phase was significantly reduced in comparison to the first phase. At the same time, the entropy was lower, and the recurrence plot showed transitions between zones less abrupt than in the first phase. This decrement of spatial dynamics, with the dispenser located in the perimeter, was more salient under the VT schedule, which was consistent with the literature on time schedules that has shown less dynamic (e.g., more time spent in zones near to the dispenser and lower distance to the dispenser) in VT than FT schedules (Van Hest et al., 1986).

In addition to the results discussed above, a low level of divergence was observed between sessions within both phases, and a high value of divergence in the transition between the last session of the first phase and the first session of the second phase was also found. This finding points to the consistency of behavioral dynamics related to each dispenser's location under each stimulus schedule and strengthen the assumptions concerning the eco-functional relevance of the different locations of the dispenser and the interaction between dispenser's location and the stimulus schedule on temporal–spatial dynamics of behavior. It is worth mentioning that the distance traveled per session (a measure of the vigor of displacement patterns) did not notably change between different locations of the dispenser. Thus, the dispenser's location did not alter the vigor of behavioral patterns, but it did alter their directionality.

The results of the current experiments expand the evidence concerning the dynamics of behavior under time-based schedules (Pear, 1985; Eldridge et al., 1988; Silva and Pear, 1995; Silva and Timberlake, 1998; Hurtado-Parrado et al., 2019) and add evidence concerning the comparison between FT and VT schedules from a parametrical framework for the analysis of the continuum of behavior by integrating different complementary dimensions related to temporal–spatial dynamics: routes, time spent in zone, distance to dispenser, recurrence plots, entropy, and divergence. These dimensions integrate a comprehensive and broad characterization of the continuum of behavior that is difficult to obtain with the approaches based only in the discrete responses or other unique measure of the spatial dimension, for example, time spent in zone (Gallistel et al., 2007; Ribes-Iñesta et al., 2018).

Our findings also suggest a high relevance of the location of the water dispenser on the dynamics of behavior that is not strictly explained in terms of the stimulus schedule and that requires integrating an ecological approach in order to explain it. The results of this study suggest that the perimeter zone maintained its ecological relevance through all sessions, in both experiments and in their two respective phases (see routes and preference plots), independently of the location of the dispenser. This ecological relevance has been extensively reported in the literature as thigmotaxis patterns (Yaski et al., 2011), “safe patterns” (Whishaw et al., 2006), and preference for closed zones (Martinez and Morato, 2004), in opposition to preference for open zones and incursions to the center area in open field situations.

Following Schneirla (1964), who pointed that approach and withdrawal patterns, that is, *towardness* or *awayness*, are the “only empirical, objective terms applicable to all behavior patterns in all animals, in order to understand how the animals manage to reach beneficial conditions and stay away from the harmful, that is, how survivors do this” (p. 511), it is plausible to say that our findings, considering *approach* and *withdrawal* patterns, show a differential ecological segmentation of the experimental arena (MOFS). From the first moment, in the first experimental session and regardless of stimuli schedule, differentiated patterns regarding closed (perimeter) vs. open (center) zones were observed: *approach* to the perimeter and *withdrawal* to the center zone. Then, in the intermediate and final session of the first phase, the functional segmentation changed: the center zone emerged as relevant segment (*towardness*), and the perimeter zone maintained its ecological relevance (*towardness*) as a safety zone. Nevertheless, segments are not qualitatively equivalent; one was a state of the space, relatively invariant (corners or walls), and the other, the center zone, was intermittent and relatively dynamic due to the fact that water appeared and disappeared in time. Therefore, the direction and preference of displacement patterns deviate and fluctuate between both segments of the environment (see graphs of distance to the dispenser), leading to a relatively high spatial dynamics of temporal–spatial behavior in the first phase in both experiments (see entropy plots).

In the second phase, both eco-functional segments, *water delivery* and *safety zone*, were contracted to the same area (perimeter area), so the majority of approach patterns were associated with this zone. In consequence, the direction and preference of the displacement patterns converge, which led to a relatively low spatial dynamics of behavior (see entropy plots).

The above explains that the spatial dynamic under VT schedule with the water dispenser located at the center of the chamber in some cases was higher than under FT schedule with the location of the dispenser in the perimeter. This would not be explained if we only take into account the well-known effect of stimuli schedules in a standard parametrical way regarding a decrement in the dynamic of behavior under VT compared to FT schedules (Ribes-Iñesta et al., 2018).

It is important to mention that, from the broad literature concerning spatial dynamics in the open field paradigm (Spruijt et al., 2014), the dynamics of behavior observed in the first phases of both experiments would seem to be unusual (e.g., displacements patterns directed and approached to the center of the open field and time spent in this zone). Nevertheless, if the modification to the open field paradigm (i.e., the occurrence of a relevant ecological event in the central area) is considered, these findings are comprehensible and invite us to reconsider the standard assumption concerning a generalized avoidance of the rats to open zones (Whishaw et al., 2006). Additionally, they show a parametrical way to endow of functional relevance the inside area of the experimental arena, in contrast with paradigms (Skinner, 1938; Ribes-Iñesta et al., 2018) that only establish functional relevance to the perimeter of the arena and, in consequence, cancel in some way the functional relevance of the space. In other words, the functional relevance of the space on the dynamics of behavior does not depend on the size of the experimental area (reduced vs. widen space) but on the relevant ecological events (or objects) that occur (or present) *inside of* or in relation with ecological differenced segments of the environment and its changes, aspect that can be referred to as *functional densification of space*.

From a systemic approach for the analysis of behavior, this is a conception of behavior as an integrated functional system comprising an *environment subsystem* and an *organism subsystem*, in which the ecological relevance of the events and segments of the space are codetermined by the qualities of the organism, defined in a phylogenetic and an ontogenetic way. Thus, the present work explored some static (e.g., delimited zones, texture path, and dispenser's location) and dynamic (e.g., water delivery and stimuli schedule) arrangements of the *environment subsystem* and its relationship with the temporal–spatial dynamics of the behavior, given a particular organism (ratus norvergicus). Any variation in relevant ecological aspects in the *environment subsystem* or in the *organism subsystem* (e.g., deprivation, alterations in sensory–perceptual systems, etc.) would have led to a different temporal–spatial dynamics of the behavior.

The present study has some limitations. One is that all subjects were exposed to the same sequence of water locations (center–wall), which could produce a sequencing effect that may have affected the results in Condition II. However, it seems implausible because, if that was the case, we would have seen that the distance to the water dispenser decreased along sessions in the first phase of the study (center); instead, figures clearly show a back-and-forth pattern, especially for the rats under the FT schedule. A second limitation is that we did not test the function of the patch as a signal of the water location. Although the fact that rats located the water source starting from session 1 in each condition suggests that the patch facilitated the contact with the water source; future studies should evaluate this.

Nonetheless, despite the potential limitations of the study, as a corollary of the present work, three contributions and future directions are worth mentioning. First, in methodological terms, the modified open field system is an alternative paradigm for different fields of study in which the analysis of the spatial dynamics in the open field are relevant, especially for studies in which the incursion to open zones or changes in the directionality of displacement patterns are relevant (Prut and Belzung, 2003; Spruijt et al., 2014). Second, and as already mentioned, an interesting issue that requires a systematic empirical analysis is the high effectiveness of the texturized patch (i.e., haptic stimulation) as a signal of the water delivery zone (see the high level of behavioral adjustment to the dispenser between phases) in rats. Finally, the present work is an example of the parsimony and heuristic value of an integrative parametrical–ecological approach for the comprehension and analysis of the temporal–spatial dynamics of behavior, which can be extended to the study of the dynamics of behavior in other species, with the respective adjustments in the environmental subsystem given certain organism subsystem.

## Data Availability Statement

The raw data supporting the conclusions of this article will be made available by the authors, without undue reservation.

## Ethics Statement

The animal study was reviewed and approved by the Animal Ethics Committee at Centro de Estudios e Investigaciones en Conocimiento y Aprendizaje Humano, Universidad Veracruzana (Protocol approval # PA-2019-01) in agreement with university regulations of animal use and care and followed the official Mexican norm NOM-062-ZOO-1999 for Technical Specification for Production, Use and Care of Laboratory Animals.

## Author Contributions

AL and VH: conceptualization, data curation, formal analysis, investigation, methodology, project administration, resources, software, supervision, visualization, roles/writing—original draft, writing—review, and editing. UH: conducted experimental sessions and data curation. CH, PT, MA, EE: data curation and formal analysis. IG: data curation and editing. All authors contributed to the article and approved the submitted version.

## Conflict of Interest

The authors declare that the research was conducted in the absence of any commercial or financial relationships that could be construed as a potential conflict of interest.
