# Supplementary material for: Ecological Location of a Water Source and Spatial Dynamics of Behavior Under Temporally Scheduled Water Deliveries in a Modified Open-Field System: An Integrative Approach
Source: Front Psychol. 2020 Dec 18;11:577903. doi: 10.3389/fpsyg.2020.577903 (PMC7775368; doi:10.3389/fpsyg.2020.577903)
Supplement: Supplementary file 1 [file Data_Sheet_1.docx]

**Supplementary Material of**

**Ecological location of a water source and spatial dynamics of behavior under temporally scheduled water deliveries in a modified open-field system: An integrative approach.**

Authors: Alejandro León^1^, Varsovia Hernández^1^, Ursula Huerta^1^; Carlos Alberto Hernández Linares^2^; Porfirio Toledo^2^; Martha Lorena Avendaño Garrido^2^, Esteban Escamilla Navarro^3^ & Isiris Guzmán^1^

^1^Comparative Psychology Lab, Universidad Veracruzana, CEICAH

^2^Universidad Veracruzana. Facultad de Matemáticas.

^3^Laboratorio Nacional de Informática Avanzada

Adress correspondence to: Alejandro León Maldonado. e-mail: [aleleon@uv.mx](mailto:aleleon@uv.mx) Centro de Estudios e Investigaciones en Conocimiento y Aprendizaje Humano (CEICAH). Agustín Melgar S/N esquina Araucarias. Col. Revolución. C.P. 91100. Xalapa, Ver. (México)

**Appendix A**

**Smoothed distance**

If $\left\{ d_{i} \right\}$ is the sequence of distance function, where the index i takes values ​​from 1 to the maximum of data N, then the values, for the smoothed distance function sequence $\left\{ \bar{d}_{i} \right\}$, are determined as follows, for a radius of K frames around each value. For $K<i<N-K$,

$$\bar{d}_{i}=\frac{1}{2K+1}\sum_{j=i-K}^{i+K} d_{j}.$$

The first K terms of $\{\bar{d}_{i}\}$, for $1\leq i\leq K$, are determined by

$$\bar{d}_{i}=\frac{1}{K+i}\sum_{j=i}^{i+K} d_{j}.$$

While the last K terms of $\{\bar{d}_{i}\}$, for $N-K\leq i\leq N$, are defined by the formula

$$\bar{d}_{i}=\frac{1}{K+N-i}\sum_{j=i-K}^{N} d_{j}.$$

**Appendix B**

**Recurrence.**

The recurrence plot is defined by symmetric matrix $A=\left[ a_{ij} \right]$ with dimensions $N\times N$, in which the inputs are determined by the following function:

$$a_{ij}=\left\{ \begin{matrix} \text{black}, & \text{if }P_{j}\in R_{k} \text{and }P_{i}\in R_{k}\text{, } \\ \text{white}, & \text{if }P_{j}\notin R_{k} \text{and }P_{i}\in R_{k}, \end{matrix} \right.$$

where $i=1,2,\ldots,N$; $j=1,2,\ldots,N$; $k=1,2,\ldots,100$. That is, the matrix $A$ (recurrence plot) is a matrix of time (frame=.2 sec) per time (frame=.2 sec), from i to j. $P_{j}$ is a given frame and $R_{k}$ is a given region (one of hundred regions). If given frames $P_{i}$ & $P_{j}$ coincide in $R_{k}$, then the value $a_{ij}=$black in the intersection between $P_{i}$ & $P_{j}$ in the matrix $A$. If given frames $P_{i}$ & $P_{j}$ do not coincide in $R_{k}$, then the value $a_{ij}=$white in the intersection between $P_{i}$ & $P_{j}$ in the matrix $A$.

**Appendix C**

**Entropy.**

The concept of entropy was introduced by Shannon (1948). Entropy is a measure associated to a discrete random variable, which indicates ‘uncertainty’ or ‘disorder’. The entropy measures uncertainty average level of the random variable. When the variable has a low-probability value, the event carries more ‘information’ than when the variable produces a high-probability value. This notion of ‘information’ is also sometimes interpreted as “surprisal".

Formally, given a discrete random variable $X$ with possible outcomes $\left\{ x_{i} \right\}$, each with probability $P\left( x_{i} \right)$, the entropy $H\left( X, P \right)$ is as follows

$$H\left( X,P \right)=-\sum P\left( x_{i} \right)ln\left( P\left( x_{i} \right) \right).$$

It can be proven that the entropy of a discrete random variable is a non-negative number, $H\left( X,P \right)\geq0$, and its measure should be maximal if all the outcomes are equally likely (uncertainty is highest when all possible events are equiprobable).

In order to analyze the displacement pattern of individuals in each session, the discrete random variables $\left\{ x_{i} \right\}$ are the permanence in each defined zone from a configuration of $10\times10$ defined zones (see Figures 5 and 12) and $P\left( x_{i} \right)$ is accumulated time (standardized) at it.

**Appendix D**

**Divergence.**

The Kullback-Leibler divergence was introduced by Kullback and Leibler (1951) and discussed by Kullback (1959). The Kullback-Leibler divergence (or relative entropy) is a measure of difference from first one probability distribution to second one.

For discrete probability distributions $P$ and $Q$ defined on the same random variable $X$, with possible outcomes $\left\{ x_{i} \right\}$, the Kullback–Leibler divergence from $Q$ to $P$ is defined as

$$D_{KL}\left( P\parallel Q \right)=\sum_{i} P\left( x_{i} \right)ln\left( \frac{P\left( x_{i} \right)}{Q\left( x_{i} \right)} \right).$$

The Kullback-Leibler divergence is defined only if for all $x$, $Q\left( x \right)=0$ implies $P\left( x \right)=0$. Whenever $P\left( x \right)$ is zero the contribution of the corresponding term is interpreted as zero.

The KL divergence $D_{KL}\left( P\parallel Q \right)$ can be thought of as something like a measurement of how far the distribution $Q$ is from the distribution $P$, because it is always non-negative ($D_{KL}\left( P\parallel Q \right)\geq0$) and a result known is $D_{KL}\left( P\parallel Q \right)$ zero if and only if $P=Q$, this is a Kullback–Leibler divergence of $0$ indicates that the two distributions in question are identical. However, it is not symmetric, that is, $D_{KL}\left( P\parallel Q \right)\neq D_{KL}\left( Q\parallel P \right)$.

To analyze the displacement pattern of individuals in consecutive sessions, the discrete random variables $\left\{ x_{i} \right\}$ are stadia in each square region from a configuration of $10\times10$ defined zones (see Figures 5 and 12) and $Q\left( x_{i} \right)$ is accumulated time (standardized) at first one session and $P\left( x_{i} \right)$ is accumulated time (standardized) at second one.

**References**

Kullback, S. (1959). *Information Theory and Statistics*. New York: John Wiley & Sons, Inc.

Kullback, S., and Leibler, R. A. (1951). On Information and Sufficiency. *The Annals of Mathematical Statistics*, 22(1), 79–86. https://doi.org/10.1214/aoms/1177729694

Shannon, C. E. (1948). A Mathematical Theory of Communication. *Bell System Technical Journal*, 27(4), 623–656. <https://doi.org/10.1002/j.1538-7305.1948.tb00917.x>
